# The Kaiser Permanente Northern California research program on genes, environment, and health (RPGEH) pregnancy cohort: study design, methodology and baseline characteristics

**DOI:** 10.1186/s12884-016-1150-2

**Published:** 2016-11-29

**Authors:** M. M. Hedderson, A. Ferrara, L. A. Avalos, S. K. Van den Eeden, E. P. Gunderson, D. K. Li, A. Altschuler, S. Woo, S. Rowell, V. Choudhary, F. Xu, T. Flanagan, C. Schaefer, L. A. Croen

**Affiliations:** Kaiser Permanente Northern California Division of Research, 2000 Broadway, Oakland, CA 94612 USA

**Keywords:** Pregnancy, Cohort, Resource, Biorepository, Maternal health

## Abstract

**Background:**

Exposures during the prenatal period may have lasting effects on maternal and child health outcomes. To better understand the effects of the in utero environment on children’s short- and long-term health, large representative pregnancy cohorts with comprehensive information on a broad range of environmental influences (including biological and behavioral) and the ability to link to prenatal, child and maternal health outcomes are needed. The Research Program on Genes, Environment and Health (RPGEH) pregnancy cohort at Kaiser Permanente Northern California (KPNC) was established to create a resource for conducting research to better understand factors influencing women’s and children’s health. Recruitment is integrated into routine clinical prenatal care at KPNC, an integrated health care delivery system. We detail the study design, data collection, and methodologies for establishing this cohort. We also describe the baseline characteristics and the cohort’s representativeness of the underlying pregnant population in KPNC.

**Methods:**

While recruitment is ongoing, as of October 2014, the RPGEH pregnancy cohort included 16,977 pregnancies (53 % from racial and ethnic minorities). RPGEH pregnancy cohort participants consented to have blood samples obtained in the first trimester (mean gestational age 9.1 weeks ± 4.2 SD) and second trimester (mean gestational age 18.1 weeks ± 5.5 SD) to be stored for future use. Women were invited to complete a questionnaire on health history and lifestyle. Information on women’s clinical and health assessments before, during and after pregnancy and women and children’s health outcomes are available in the health system’s electronic health records, which also allows long-term follow-up.

**Discussion:**

This large, racially- and ethnically-diverse cohort of pregnancies with prenatal biospecimens and clinical data is a valuable resource for future studies on in utero environmental exposures and maternal and child perinatal and long term health outcomes. The baseline characteristics of RPGEH Pregnancy Cohort demonstrate that it is highly representative of the underlying population living in the broader community in Northern California.

**Electronic supplementary material:**

The online version of this article (doi:10.1186/s12884-016-1150-2) contains supplementary material, which is available to authorized users.

## Background

Exposures before and during pregnancy contribute to the immediate and future health outcomes of both women and their children. Emerging evidence supports the notion that the prenatal period is a critical developmental window during which in utero exposures may have lasting effects on a child’s future health [1, 2]. Biological programming [3] occurs during fetal life in response to in utero exposure to nutrient substrates, hormones, growth factors, cytokines, environmental conditions or toxins, and other exposures. Evidence also shows that women who develop pregnancy complications are at increased risk of developing chronic diseases later in life [4–7]. However, the mechanisms underlying many of these findings remain unclear, and further research is needed to advance our understanding of how the in utero environment impacts the short- and long-term health of both the woman and her child.

Large studies with multiple measurements of biomarkers during pregnancy are needed to better measure perinatal exposures and to understand the etiologically relevant period of the effects of exposures on perinatal outcomes. To fully understand how the in utero environment influences the short- and long-term health of women and their children, large representative study populations with comprehensive information on a broad range of factors, including biomarkers, medical conditions, medications, nutrition, physical activity and environmental exposures, are needed.

The Kaiser Permanente Northern California (KPNC) Research Program on Genes, Environment, and Health (RPGEH) has established a large pregnancy cohort that integrates biospecimens with rich and accurate clinical and health data available from the electronic health record (EHR), creating a unique resource available to advance research on women’s and children’s health. The establishment of this pregnancy cohort within an integrated health care delivery system with an EHR has the additional advantage of enabling accurate assessment of short- and long-term maternal and child health outcomes and the rapid translation of clinically meaningful findings into clinical practice. This report describes the design and methods used to establish this pregnancy cohort and its biorepository in KPNC. We present preliminary data on the baseline characteristics of the cohort to demonstrate its racial-ethnic diversity and the prevalence of several perinatal complications of interest, as well as its representativeness with regard to the underlying population of pregnancies at KPNC. We further discuss possible use of this large cohort including the ability to efficiently follow it prospectively through the EHR to answer pressing questions regarding women’s and children’s health.

## Methods/Design

### Aim

The aim of this project is to establish a large pregnancy cohort that integrates biospecimens with rich and accurate clinical and health data to create a resource to advance scientific research on women’s and children’s health. The pregnancy cohort is able to be linked to short- and long-term maternal and child health outcomes to facilitate the rapid translation of clinically meaningful findings into clinical practice.

### Design

The KPNC Division of Research started the Research Program on Genes, Environment and Health (RPGEH) in 2007 to develop a genetic epidemiology population resource which integrates data from multiple sources from consenting KPNC adult members, including biospecimens, clinical data from the EHR, lifestyle and risk factor data from surveys, and environmental exposure data from both laboratory and geographic information systems. One component of the RPGEH is the RPGEH Pregnancy Cohort.

### Establishment of RPGEH pregnancy cohort

The Division of Research worked closely with KPNC clinical partners to develop facility-based recruitment procedures and laboratory blood processing workflows that could be easily integrated as part of routine prenatal medical care. The entire recruitment process was designed to become an integrated and routine part of the clinical prenatal intake process. To avoid disruption of clinical workflows, all RPGEH program-related processes (e.g., questions from patients, and follow-up) are handled by research staff. The recruitment and biospecimen collection protocol processes are described below.

### Study setting

KPNC provides integrated health care to over 3.6 million members through 7,000 physicians, > 240 medical office buildings and 22 hospitals. The KPNC service area spans 14 counties of the greater Bay Area, as well as the California Central Valley from Sacramento to Fresno and includes urban and rural areas. The population is highly representative of the demographic characteristics of the entire population from this geographic area [8]. The membership is racially and socio-economically diverse. KPNC is vertically integrated such that all care is provided in a closed system and documented in an EHR. The EHR are clinical records, not claims data, and thus are robust with regard to data quality and completeness. The membership of reproductive-aged women (15–44) includes women with KP commercial insurance (varying copays, varying deductible levels), MediCal, and other California state subsidized programs. Within KPNC, there are 16 delivery hospitals and approximately 38,000 pregnancies each year.

### Recruitment of participants

**Fig. 1:**
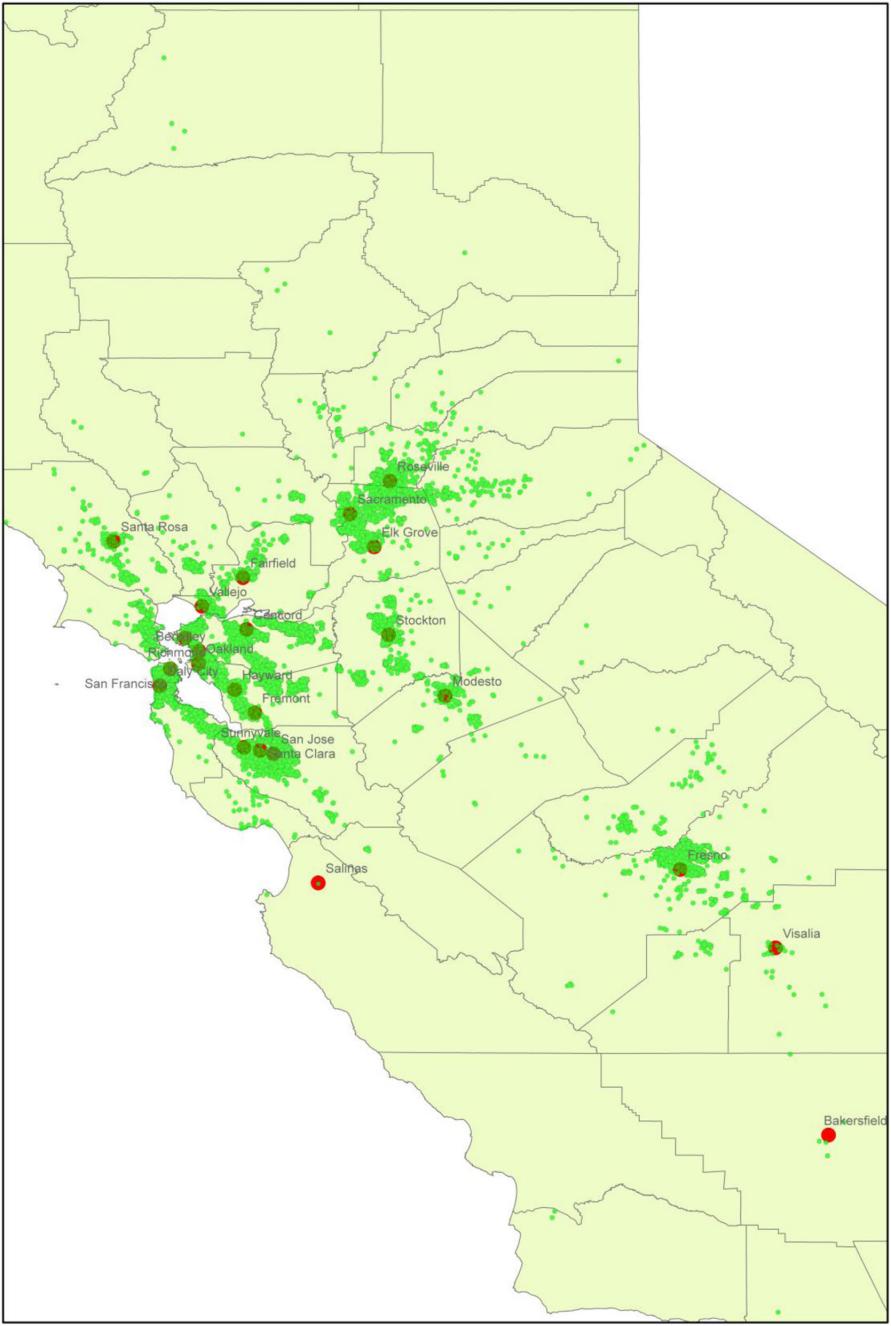
Geographic locations of Kaiser Permanente Northern California RPGEH Pregnancy Cohort members

The RPGEH Pregnancy Cohort recruitment began in February 2010 at the KPNC Walnut Creek outpatient medical facility. Recruitment gradually expanded to cover almost the entire KPNC service area. Figure 1 shows the geographical locations of RPGEH pregnancy cohort members in an area of over 28,000 square miles, an area slightly larger than South Carolina. Clinical staff, such as medical assistants and nurses at the Obstetrics and Gynecology department, routinely gives a RPGEH pregnancy cohort flyer with frequently asked questions and a consent form to women at the initial prenatal visit. They also briefly describe the RPGEH Pregnancy Cohort and ask women if they would like to participate. If the woman agrees to participate and signs the consent form, the clinic staff places the research blood draw order in the woman’s EHR.

### Biospecimen collection and storage process

Women who consent have blood drawn for research purposes into one 8.5 mL serum separator tube (SST) tube and one 6.0 mL ethylenediaminetetraacetic Acid (EDTA) tube at the same time as the clinically ordered blood tests at her local KPNC laboratory at two times during their pregnancy: in the first trimester during a standard first trimester panel or genetic screening (~10–13 weeks, 6 days) and during the second trimester either along with standard genetic screening (~15–20 weeks) or with the gestational diabetes screening (~24–28 weeks). The blood tubes are couriered as part of the normal KPNC laboratory system to the Regional Laboratory, where they are transferred to the RPGEH Biorepository (see description below) and further processing occurs.

### The RPGEH Research Biorepository

The RPGEH Biorepository is a state-of-the-art research biorepository and staffed with research laboratory personnel who are responsible for maintaining the laboratory space, checking in, processing and storing samples, and retrieving aliquots for studies. Equipment includes an ABF 500 automated blood fractionation robot unit, an RTS A4 temperature and humidity controlled robotic ambient storage unit for archiving DNA using Biomatrica DNA stable storage medium, and a walk-in−80 ° C freezer. A custom developed Laboratory Information Management System (LIMS) tracks specimens at each step and is linkable to RPGEH operations and clinical information databases.

Once at the Biorepository, serum from the SST is aliquotted into 4, 0.8 mL cryovials. The EDTA tube is centrifuged and plasma is aliquoted into 2, 0.8 mL cryovials, while 1.0 mL of buffy coat is aspirated and placed in a cryovial. All cryovials are stored at−80 °C.

### Clinical data

Information on participants in the Pregnancy Cohort is obtained from several sources of rich clinical data (resources are described below).

### Information obtained from the EHR during the first prenatal visit

As part of routine prenatal care, all pregnant women complete a prenatal questionnaire during the first trimester or shortly after the pregnancy is clinically confirmed. This questionnaire includes questions on parity, gravidity, prior delivery and birth history, reproductive history, menstrual history, prior medical history, social circumstances (e.g., stress, domestic violence, etc.), and an Adult Outcomes Questionnaire (AOQ) which includes the PHQ-9 [9, 10] depression screener and the Generalized Anxiety Disorder scale (GAD-2) [11] as well as functioning items. The information from the Prenatal Questionnaire is recorded in the KPNC EHR for access to extensive health and reproductive history on the cohort. Several other sources of pre-pregnancy information are available in the EHR including pre-pregnancy body mass index (BMI) if a woman had been a KPNC member prior to conception.

### Early start substance use data

In addition to the Prenatal Questionnaire, a self-administered Early Start Program Prenatal Substance Use Screening Questionnaire is completed at entry into prenatal care. Early Start is an integrated prenatal program to intervene when a pregnant woman reports alcohol, tobacco and other drug use during pregnancy [12]. The questionnaire asks about substance use before pregnancy and since pregnancy began, including alcohol, smoking, and prescription drug use.

### Clinical data available in KPNC EHR

KPNC maintains complete databases that capture all encounters including hospitalizations, outpatient visits, radiology/imaging, laboratory tests, and prescription medications and combines these data for presentation to clinicians as part of the EHR. Data captured in these databases include inpatient and outpatient diagnostic information, imaging reports, laboratory tests and results, pharmacy dispenses including dosages and days of supply, and surgery outcomes, among others. All vital signs including weight and height, blood pressure and physical activity are recorded in the EHR. As noted above, these data are clinical information maintained in an EHR and are not claims data, and enable the detailed examination of diagnoses and treatments before, during and after pregnancy. In addition, when an infant is born, he/she is issued a unique medical record number (MRN) that is used for all care associated with the infant. It is linkable to the mother’s unique MRN that allow identification of the mother-infant pair. This allows us to also link to the women’s infants and examine infant growth and outcomes at birth and during childhood, along with other health outcomes, including the mother’s outcomes.

### RPGEH pregnancy cohort questionnaire

To obtain more detailed information not captured in the HER, each participant is invited to complete the RPGEH Pregnancy Cohort questionnaire. The RPGEH questionnaire ascertains information about a variety of socio-demographic, lifestyle and environmental factors not routinely captured in the EHR, including diet, physical activity, multivitamin use and self-reported health history before and during the study pregnancy (Additional file 1).

### Environmental exposure data

Over 98 % of the RPGEH pregnancy cohort has been successfully geocoded and can be linked to contextual or environmental data, including spatiotemporal data that exist in public access databases. These data come from commercial sources, non-profit agencies, and local, regional, state and national government agencies. Data from these various sources are being incorporated into a KPNC geographic information system (GIS) database using ArcGIS software (Redlands, CA). The database will include data on retail food outlets, green space, infrastructure (roads, educational facilities, health delivery centers, and public assistance facilities), traffic density, air pollution, pesticide use, toxic sites, toxic release inventories, and other factors. Other relevant information, currently located at other agencies but available for linkage, includes water quality, centers of social congregation (e.g., religious or spiritual institutions, senior centers, youth activity centers, etc.), and crime data. California has some of the most complete publically available geospatial data across these environmental factors anywhere in the world.

Below we describe the sources used for determining the clinical outcomes of the RPGEH Pregnancy Cohort participants and non-participants for this preliminary report.

### Clinical outcomes during pregnancy/in utero exposure to maternal metabolism

#### Women’s body mass index and gestational weight gain

Through the EHR we are able to capture a woman’s body mass index prior to pregnancy as well her gestational weight gain trajectory and total gestational weight gain, allowing us to assess possible determinants of gestational weight gain, as well as to define the sequelae of in utero exposure to maternal obesity and excessive gestational weight gain (i.e., over nutrition) or inadequate gestational weight gain (i.e., undernutrition) in relation to the current Institute of Medicine guidelines [13] on child health.

#### Pregestational diabetes and gestational diabetes mellitus (GDM) and impaired glucose tolerance

Pregestational diabetes is obtained from the KPNC Diabetes Registry [14] and GDM is obtained from the KPNC pregnancy glucose tolerance and GDM Registry [15]. These registries allow for the identification of GDM based on objective glucose measurement defined according to laboratory glucose values meeting the Carpenter and Coustan diagnostic criteria [16].

#### Preeclampsia/Hypertensive disorder of pregnancy

Preeclampsia and hypertensive disorders of pregnancy were also obtained from the EHR and were defined according to the following ICD-9 codes: pre-existing hypertension 642.0–642.2, gestational hypertension 642.3, preeclampsia or eclampsia 642.4–642.7. The validity of these ICD-9 codes to diagnose hypertensive disorders of pregnancy has previously been reported [17].

### Clinical outcomes at birth

#### Preterm birth

Gestational age is based on the estimated date of delivery recorded in the EHR, which is determined by the woman’s self-reported last menstrual period (LMP), or by first trimester ultrasound if different from the LMP-based calculation by more than 1 week. Preterm birth was defined as birth at <37 weeks’ gestation. We also examined the degree of preterm birth using the following definitions: extreme preterm (<28 weeks’ completed gestation), severe preterm (28–31 weeks' completed gestation), moderate preterm (32–33 weeks' completed gestation) and late preterm (34–36 weeks' completed gestation) [18].

#### Infant size for gestational age

Infant birthweight was obtained from the EHR. Large for gestational age was defined as birthweight >90th percentile and small for gestational age was defined as birthweight <10th percentile for the underlying KPNC population’s race-ethnicity and gestational age–specific birthweight distribution [19].

#### Cesarean delivery

Cesarean delivery information was obtained from the KPNC neonatal and infant cohort [20] and is defined according to ICD-9 codes 654.2× for delivery mode recorded in the EHR.

### Recruitment to date and prevalence of outcomes of interest

Between February 2010 and October 2014, pregnant members of KPNC aged 18 or older who initiated prenatal care at a KPNC medical facility participating in the pregnancy cohort were invited to participate in the RPGEH pregnancy cohort. Among the 93,409 pregnancies occurring at medical facilities participating in the RPGEH pregnancy cohort during this initial recruitment period, 16,977 RPGEH pregnancy cohort consent forms were received, which represents a participation rate of 18.2 %. Compared to non-participants, women who participated in the pregnancy cohort were similar in age, but were more likely to have initiated prenatal care in the first trimester and to be non-Hispanic white and were slightly less likely to be Asian (Table 1). RPGEH pregnancy cohort participants were KPNC members for an average of 10 years before their pregnancy (Table 1). Among the 16,977 RPGEH pregnancy cohort participants who delivered a liveborn infant at the time of this writing, 93.2 % had a first trimester blood draw (mean gestational age: 9.1 weeks +/−4.2 SD) and 80.5 % had a second trimester blood draw (mean gestational age: 18.1 weeks +/−5.5 SD) and 77.6 % had blood drawn in both trimesters.

**Table 1:** Characteristics of RPGEH Prenatal Cohort Participants Compared to Non-Participants in Kaiser Permanente Northern California

	Overall Denominator (*n* = 93,409)	Participants (*n* = 16,977)	Non-Participants (*n* = 76,432)
Characteristic	N (%)		
Age^a^			
18–24	14,590 (15.6)	2,456 (15.1)	12,134 (15.9)
25–29	25,685 (27.5)	4,769 (28.1)	20,916 (27.4)
30–34	32,469 (34.8)	6,158 (36.3)	26,311 (34.4)
35–39	16,574 (17.7)	2,940 (17.9)	13,634 (17.8)
40 or older	4,065 (4.4)	653 (3.8)	3,412 (4.5)
Unknown	26 (<0.1)	1 (<0.1)	25 (<0.1)
Race/Ethnicity			
Non-Hispanic White	36,704 (39.3)	7,975 (47.0)	28,729 (37.6)
Hispanic	22,578 (24.2)	3,909 (23.0)	18,669 (24.4)
Asian	22,413 (24.0)	3,264 (19.2)	19,149 (25.0)
African American	6,448 (6.9)	960 (5.6)	5,488 (7.2)
Other	3,965 (4.2)	691 (4.1)	3,274 (4.3)
Unknown	1,301 (1.4)	178 (1.1)	1,123 (1.5)
Parity			
0	58,395 (62.5)	10,312 (60.7)	48,083 (62.9)
1	26,210 (28.1)	5,030 (29.6)	21,180 (27.7)
2+	8,804 (9.4)	1,635 (9.6)	7,169 (9.4)
Pre-pregnancy BMI (kg/m^2^)			
< 18.5	1919 (2.4)	276 (1.9)	1,643 (2.5)
18.5–24.9	36,957 (46.2)	6,607 (44.8)	30,350 (46.5)
25.0–29.9	20, 758 (25.9)	4,013 (27.2)	16,745 (25.6)
30.0+	17,132 (21.4)	3546 (24.0)	13,586 (20.8)
Unknown	3,320 (4.2)	315 (2.1)	3,005 (4.6)
Gestational age at initiation of prenatal care			
1st trimester	83,551 (89.5)	16,041 (94.5)	67,510 (88.3)
2nd trimester	6,707 (7.2)	722 (4.3)	5,985 (7.8)
3rd trimester	2,980 (3.2)	141 (0.8)	2,839 (3.7)
Unknown	171 (0.2)	73 (0.4)	98 (0.1)
Length of enrollment before pregnancy (years)	8.9 (8.6)	10.0 (9.1)	8.6 (8.5)
Length of enrollment after pregnancy (years)	2.5 (1.2)	2.7 (1.3)	2.5 (1.2)

^a^Age at standard first trimester prenatal panel blood draw

Of the 93,409 pregnancies initially identified, 80,086 (84 %) delivered an infant in Kaiser Permanente Northern California. Of the pregnancies not resulting in livebirths, 5.7 % were due to pregnancy loss, 4.6 % no longer had Kaiser medical coverage, and 4.0 % delivered outside of Kaiser. Among the deliveries in Kaiser Permanente Northern California, the prevalence of preterm birth (<37 weeks), cesarean delivery, small for gestational age, large for gestational age, macrosomia, preeclampsia, GDM and NICU admissions was similar between RPGEH pregnancy cohort participants and non-participants (none of these outcomes differed by more than 1.2 %; see Table 2).

**Table 2:** Perinatal Outcomes of the RPGEH Prenatal Cohort Participants Compared to Non-Participants in Kaiser Permanente Northern California

	Overall Denominator (*n* = 80,086)	Participants (*n* = 14,757)	Non-Participants (*n* = 65,329)
Perinatal Outcome		N (%)	
Preeclampsia			
None	71,368 (89.1)	13,017 (88.2)	58,315 (89.3)
Pre-existing hypertension	2,441 (3.1)	524 (3.4)	1,917 (2.9)
Gestational hypertension	3,079 (3.8)	604 (4.1)	2,475 (3.8)
Preeclampsia/Eclampsia	3,198 (4.0)	612 (4.1)	2,586 (4.0)
Glucose Tolerance Status			
Normal Screening	56,461 (70.5)	10,861 (73.6)	45,599 (69.8)
Abnormal Screening	12,013 (15.0)	2,125 (14.4)	9,930 (15.2)
Gestational Diabetes	5,366 (6.7)	959 (6.5)	4,508 (6.9)
Preexisting T2DM	961 (1.2)	162 (1.1)	784 (1.2)
Not Screened	4,805 (6.4)	649 (4.4)	4,508 (6.9)
Gestational Age			
< 37 weeks	6,063 (7.6)	1,095 (7.4)	4,968 (7.6)
≥ 37 weeks	74,005 (92.4)	13,662 (92.6)	60,343 (92.4)
Unknown	18 (<0.1)	0 (0)	18 (<0.1)
Gestational Age			
Extreme Preterm (<28 weeks)	324 (0.4)	57 (0.4)	267 (0.4)
Severe Preterm (28–31 week)	526 (0.7)	91 (0.6)	435 (0.7)
Moderate Preterm (32–33 weeks)	698 (0.9)	116 (0.8)	582 (0.9)
Late Preterm (34–36 weeks)	4,515 (5.6)	831 (5.6)	3,684 (5.6)
≥ 37 weeks	74,005 (92.4)	13,662 (92.6)	60,343 (92.4)
Unknown	18 (<0.1)	0 (0)	18 (<0.1)
Gestational weight gain in relation to the IOM weight gain guidelines			
Below	10,456 (13.1)	1,847 (12.5)	8,609 (13.2)
Met	17,196 (21.5)	3,115 (21.1)	14,081 (21.6)
Exceeded	48,689 (60.8)	9,416 (63.8)	39,273 (60.1)
Unknown	3,745 (4.7)	379 (2.6)	3,366 (5.2)
Infant birth weight (grams)^a^	3,361 ± 556	3,400 ± 565	3,350 ± 553
AGA	63,843 (81.4)	11,771 (81.3)	52,072 (81.4)
SGA	7,436 (9.5)	1,339 (9.3)	6,097 (9.5)
LGA	6,992 (8.9)	1,342 (9.3)	5,650 (8.8)
Unknown	135 (0.2)	17 (0.1)	118 (0.2)
Infant birth weight (grams)			
Very low birth weight (<1,500)	605 (0.8)	106 (0.7)	499 (0.8)
Low birth weight (1,500–2,500)	3,158 (4.0)	555 (3.8)	2,603 (4.1)
Normal birth weight (2,501–3,999)	65,975 (84.2)	12,042 (83.2)	53,933 (84.4)
Macrosomia (4,000+)	8,582 (10.9)	1,757 (12.2)	6,825 (10.7)
Unknown	86 (0.1)	9 (0.1)	77 (0.1)
NICU Stay			
Non-NICU	71,563 (89.4)	13,092 (88.7)	58,471 (89.5)
NICU	8,393 (10.5)	1,647 (11.2)	6,746 (10.3)
Delivery room death	112 (0.1)	17 (0.1)	95 (0.2)
Unknown	18 (<0.1)	1 (<0.1)	17 (<0.1)

^a^LGA is large-for-gestational age, adjusted for race/ethnicity

SGA is small-for-gestational age, adjusted for race/ethnicity

AGA is appropriate for-gestational age, adjusted for race/ethnicity

Participants were slightly more likely to be screened for GDM (95.6 % versus 93.1 %). Overall, participants and non-participants were very similar in their behavioral risk factors assessed on the Early Start Questionnaire at the first prenatal visit (Table 3). Participants and non-participants did not differ in terms of smoking during the 12 months before pregnancy or during pregnancy. However, participants were slightly more likely to report drinking alcohol both before pregnancy (Table 3).

**Table 3:** Substance use before and during pregnancy among RPGEH Prenatal Cohort Participants compared to Non-Participants in Kaiser Permanente Northern California

	Overall Denominator (*n* = 87,928)	Participants (*n* = 16,584)	Non-Participants (*n* = 71,344)
Early Start Data		N (%)	
Smoking in 12 Months before Pregnancy			
Never	78,710 (89.5)	14,683 (88.5)	64,027 (89.7)
Monthly or Less	2,944 (3.4)	623 (3.8)	2,321 (3.3)
Weekly	1,304 (1.5)	230 (1.4)	1,074 (1.5)
Daily	4,747 (5.4)	1,016 (6.1)	3,731 (5.2)
Unknown	223 (0.2)	32 (0.2)	191 (0.3)
Smoking since Pregnancy			
Never	85,166 (96.9)	16,010 (96.5)	69,156 (96.9)
Monthly or Less	789 (0.9)	159 (1.0)	630 (0.9)
Weekly	537 (0.6)	117 (0.7)	420 (0.6)
Daily	1,196 (1.3)	267 (1.6)	929 (1.3)
Unknown	240 (0.3)	31 (0.2)	209 (0.3)
Alcohol in 12 Months before Pregnancy			
Never	28,267 (32.2)	4,202 (25.3)	24,065 (33.7)
Monthly or less	39,589 (45.0)	7,908 (47.7)	31,681 (44.4)
Weekly	18,102 (20.6)	4,058 (24.5)	14,044 (19.7)
Daily	1,429 (1.6)	327 (2.0)	1,102 (1.6)
Unknown	541 (0.6)	89 (0.5)	452 (0.6)
Alcohol since Pregnancy			
Never	78,796 (89.6)	14,551 (87.7)	64,245 (90.1)
Monthly or Less	6,780 (7.7)	1,517 (9.2)	5,263 (7.3)
Weekly	1,423 (1.6)	344 (2.1)	1,079 (1.5)
Daily	179 (0.2)	34 (0.2)	145 (0.2)
Unknown	750 (0.9)	138 (0.8)	612 (0.9)
Use of Prescription Drug in 12 Months before Pregnancy^a^			
No	71,212 (81.0)	13,067 (78.8)	58,145 (81.5)
Yes	16,066 (18.3)	3,403 (20.5)	12,663 (17.7)
Unknown	650 (0.7)	114 (0.7)	536 (0.8)
Use of Prescription Drug since Pregnancy			
No	83,680 (95.2)	15,726 (94.8)	67,954 (95.3)
Yes	3,871 (4.4)	799 (4.8)	3,072 (4.3)
Unknown	377 (0.4)	59 (0.4)	318 (0.4)
Use of Illegal Drug in 12 Months before Pregnancy^b^			
No	79,954 (90.9)	14,790 (89.1)	65,164 (91.3)
Yes	7,149 (8.1)	1,506 (9.1)	5,643 (7.9)
Unknown	825 (1.0)	288 (1.8)	537 (0.8)
Use of Illegal Drug since Pregnancy			
No	85,202 (96.9)	15,878 (95.7)	69,324 (97.2)
Yes	2,033 (2.3)	414 (2.5)	1,619 (2.3)
Unknown	693 (0.8)	292 (1.8)	401 (0.5)

^a^Prescription drugs in Early Start data include anxiety medications, pain medications, and sleep medications

^b^Illegal drugs in Early Start data include Cocaine, Heroin, Marijuana, and Methamphetamines

The use of RPGEH Pregnancy Cohort specimens and data are governed by the guiding principles of use and access established by the RPGEH. These principles include: 1) promote good science for the benefit of the public; 2) protect participant confidentiality and privacy; honor commitments made to participants and act within the scope of their consent; and preserve the trust that KPNC members have in KPNC; 3) comply with applicable legal and regulatory requirements; 4) consider whether the Resource is the best or only resource to address proposed research questions; 5) conserve limited materials or resources for high-value research, such as biospecimens, which can be exhausted, and use of biospecimens that are rare or of higher value because of the data associated with them; 6) ensure that an investigator at the KPNC Division of Research (DOR) is involved in the research question and the conduct of the study to ensure the right and appropriate use of the resources. Applications for use of RPGEH Pregnancy Cohort samples and data are submitted and reviewed by the RPGEH Access Review Committee (ARC). The ARC meets three times a year to review applications for use of RPGEH data and specimens. The ARC includes DOR investigators, plus external stakeholders and investigators to address specific content and methodological issues as required by the projects under consideration. The ARC governs access to and use of all RPGEH data and specimens by requestors. Applications for access will be subject to three phases of review, and the ARC’s decisions are made based on a formalized set of criteria that can be reviewed.

### Statistical analyses, power and sample size considerations

Based on our expected cohort size of 25,000 women we computed power for hypothetical case-control studies. We assumed all available cases will be included and controls will be sampled at a ratio of 5:1. We computed the minimum detectable odds ratio (OR) for a two-sided test at level 0.05 and 80 % power for several outcomes with different prevalences. For the outcome of small for gestational age (prevalence 9.3 %) a case-control study will be powered to detect an OR of 1.15. For the outcome of gestational hypertension (prevalence 4.1 %) a case-control study will be powered to detect an OR of 1.22. For the rare outcome of very low birthweight (prevalence 0.7 %) a case-control study will be powered to detect an OR of 1.57.

## Discussion

This report provides a brief overview of the establishment of the KPNC RPGEH Pregnancy Cohort and its biorepository, which were created to provide a resource for women’s and children’s health research. The KPNC RPGEH Pregnancy Cohort is uniquely integrated into routine clinical prenatal care within the KPNC health care system setting and can be linked with data from the EHR. KPNC contains a racially and ethnically diverse population, thereby increasing the likelihood of obtaining a highly representative sample with generalizable findings.

The establishment of this valuable resource has the potential to address many key questions related to women’s and children’s health and is particularly timely, in light of the recent dissolution of The National Children’s Study. The National Children’s Study (NCS) was developed after a 1990s White House Task Force highlighted the paucity of evidence evaluating the links between environmental exposures, development, and health outcomes in children and adults. The Children’s Health Act of 2000 initiated the conduct of a national longitudinal study of environmental influences (including physical, chemical, biological, and psychosocial) during pregnancy on child health and development. A recent report explains that this study was dissolved due to feasibility and oversight issues [21, 22] and suggests that funding agencies support smaller focused studies designed as tailored explorations as well as cohorts to facilitate longitudinal biospecimen collection and banking.

This large pregnancy cohort, derived from a diverse base population, can be used to generate sets of cases and controls for future clinical research studies, as demonstrated by our preliminary data. The availability of rich clinical data from the EHR, the questionnaire data, and existing perinatal research programs provide detailed phenotypic information that will further facilitate the conduct of perinatal epidemiology and translational studies. The RPGEH Pregnancy Cohort, coupled with the state of the art KPNC Biorepository for long-term storage of serum, plasma and DNA samples and an ability to follow both women and their child long term for future health outcomes in the EHR, provides a truly unique and valuable resource for improving our understanding of women and children’s health.

Our preliminary data on the RPGEH Pregnancy Cohort demonstrate that at least 18.2 % of pregnant women participated, and the cohort is highly representative of the underlying KPNC pregnant population in terms of both maternal demographics and key perinatal outcomes. Pregnancy cohort participants were KNPC members on average 10 years before their index pregnancy and remained members on average 2.7 years after pregnancy to date, and most are still currently KPNC members. Thus, there is a unique ability to examine exposures even years before pregnancy and to follow women and their infants for years after delivery. While participating women were slightly more likely to be non-Hispanic white and less likely to be Asian, this pattern is frequently observed in cohort studies with multiethnic populations such as KPNC women of reproductive age. Overall, the RPGEH Pregnancy Cohort is extremely diverse, with 53 % of participants from non-white racial ethnic minority groups, and Asian women comprise 23 % of the cohort. This is especially significant as Asian women have previously been reported as less likely to participate in reproductive and biospecimen research [22, 23]. The racial-ethnic diversity of this population provides important potential for studies examining racial-ethnic disparities in diseases and health care delivery. Given the recruitment efforts integration within clinical care, it is possible that not all pregnant women at participating medical facilities were invited to participate in the pregnancy cohort; therefore, 18.2 % is likely an underestimate of the overall participation rate.

The prevalence of several perinatal complications was similar between RPGEH cohort participants and the underlying populations of women delivering in KPNC. Cohort participants were slightly less likely to have gestational diabetes mellitus (GDM) and infants of participants were slightly more likely to be macrosomic relative to non-participants. The lower prevalence of GDM among RPGEH participants is probably due in part to the fact that participants were less likely to be Asian and more likely to be non-Hispanic white; in this setting, Asian woman have the highest prevalence of GDM [15, 24] and non-Hispanic white women have the lowest prevalence of GDM.

The fetal origins of adult disease hypothesis posits that “fetal programming” occurs when maternal metabolic nutrition, environment and hormonal milieu during development permanently programs the structure and physiology of organs and hence the future health of the offspring [25]. While there is some epidemiologic evidence supporting the “fetal programming” hypothesis, more longitudinal, observational studies examining the effects of a broad range of environmental and biological factors assessed in utero are needed to clarify the extent to which fetal programming contributes to adult diseases. In addition, a woman’s health status during pregnancy may also influence her future health [26]. For example, women diagnosed with pregnancy-related hypertension and/or preeclampsia, gestational diabetes and preterm birth are at higher risk for hypertension, diabetes and cardiovascular disease later in life [7]. Therefore, given the rich health data in the KPNC EHR, the RPGEH Pregnancy Cohort will also allow for a lifecourse research approach [27].

The resource is available to be used by Kaiser Permanente researchers as well as outside investigators who wish to collaborate with a Kaiser Permanente researcher to conduct biomarker, genetic, environmental and gene environment interaction studies. The RPGEH Pregnancy Cohort has the unique ability to connect biospecimens collected at two time points during pregnancy with detailed short- and long-term environmental and clinical data on both women and their children, enabling research of immediate perinatal complications as well as longer term maternal, child, and adult outcomes.

## Additional files

Additional file 1: Appendix 1. PG En Survey 2012-02-17 Pregnancy Cohort survey. (DOCX 33 kb)

## Abbreviations

ARC: Access review committee
DOR: Division of Research
EDTA: Ethylenediaminetetraacetic acid
EHR: Electronic health record
GDM: Gestational diabetes mellitus
KPNC: Kaiser Permanente Northern California
LMP: Last menstrual period
NCS: National children’s study
RPGEH: Research Program on Genes Environment and Health
SST: Serum separator tube
