# Supplementary material for: The Kaiser Permanente Northern California research program on genes, environment, and health (RPGEH) pregnancy cohort: study design, methodology and baseline characteristics
Source: BMC Pregnancy Childbirth. 2016 Nov 29;16:381. doi: 10.1186/s12884-016-1150-2 (PMC5129213; doi:10.1186/s12884-016-1150-2)
Supplement: Additional file 1: Appendix 1. — PG En Survey 2012-02-17 Pregnancy Cohort survey. (DOCX 33 kb) [file 12884_2016_1150_MOESM1_ESM.docx]

1. **What is your date of birth?**

🞎🞎/ 🞎🞎 / 🞎🞎🞎🞎

MO / DAY / YEAR

**2. Where you born in the United States?**

🞏_1_ No 🞏_2_ Yes 🞏_3_ Don’t know

**3. Were you born at a Kaiser Permanente hospital?**

🞏_1_ No

🞏_2_ Yes, Northern California

🞏_3_ Yes, other region

🞏_4_ Don’t know

**4. Are you a twin or triplet?**

🞏_1_ No

🞏_2_ Yes, I am a twin

🡺 🞏_1_ Identical 🞏_2_ Fraternal 🞏_3_ Don’t know

🞏_3_ Yes, I am a triplet

**5. What best describes your race or ethnicity?**

*Mark all groups that apply to you.*

🞏_1_ African-American

🞏_2_ African

🞏_3_ Afro-Caribbean

🞏_4_ Mexican

🞏_5_ Central/South American

🞏_6_ Puerto Rican

🞏_7_ Cuban

🞏_8_ Other Latin**a**/Hispanic

🞏_9_ South Asian (Indian, Pakistani, etc.)

🞏_10_ Chinese

🞏_11_ Japanese

🞏_12_ Korean

🞏_13_ Filipin**a**

🞏_14_ Vietnamese

🞏_15_ Other Southeast Asian (Cambodian, Laotian, etc.)

🞏_16_ Native Hawaiian

🞏_17_ Samoan

🞏_18_ Other Pacific Islander

🞏_19_ Native American Indian or Alaska Native

🞏_20_ White or European-American

🞏_21_ Middle Eastern

🞏_22_ Ashkenazi Jewish

🞏_23_ Other (please specify): ____________

🞏_24_ Don’t know

**6. Was your mother born in the United States?**

🞏_1_ No 🞏_2_ Yes 🞏_3_ Don’t know

**7. Was your father born in the United States?**

🞏_1_ No 🞏_2_ Yes 🞏_3_ Don’t know

**8. What is your religious background?**

🞏_1_ Buddhist 🞏_5_ Protestant

🞏_2_ Catholic 🞏_6_ Muslim

🞏_3_ Hindu 🞏_7_ None

🞏_4_ Jewish 🞏_8_ Other (please specify):__________

**9. What is the highest level of school that you have completed?**

🞏_1_ Grade school (grades 1-8)

🞏_2_ Some high school (grades 9-11)

🞏_3_ High school or GED

🞏_4_ Technical/trade school

🞏_5_ Some college

🞏_6_ College

🞏_7_ Postgraduate degree

🞏_8_ Other: ____________

**10. What is your employment or work status?**

*Mark all that apply.*

🞏_1_ Full-time employed 🞏_5_ Full-time student

🞏_2_ Part-time employed 🞏_6_ Homemaker

🞏_3_ Retired 🞏_7_ Unemployed

🞏_4_ Disabled 🞏_8_ Other (specify):__________

**11. What is your current marital status?**

🞏_1_ Never married 🞏_4_ Divorced

🞏_2_ Married,or living as married 🞏_5_ Widowed

🞏_3_ Separated

**12. Which of the following best describes you?**

🞏_1_ Heterosexual, straight 🞏_3_ Bisexual

🞏_2_ Homosexual, lesbian 🞏_4_ Other (specify): __________

**13. How many other people live in your household (include spouse, partner, children, and other relatives)?**

🞏_1_ Live alone 🞏_2_ One 🞏_3_ Two 🞏_4_ Three 🞏_5_ Four or more

**14. What best describes your household income (before taxes)?**

🞏_1_ Less than $10,000/year 🞏_5_ $40,000 – $59,999/year

🞏_2_ $10,000 – $14,999/year 🞏_6_ $60,000 – $99,999/year

🞏_3_ $15,000 – $19,999/year 🞏_7_ $100,000 – $199,999/year

🞏_4_ $20,000 – $39,999/year 🞏_8_ $200,000 or more/year

**15. In general, how would you describe your health?**

🞏_1_ Excellent 🞏_4_ Fair

🞏_2_ Very Good 🞏_5_ Poor

🞏_3_ Good

**16. What is your height without shoes?**

🞏_1_ Feet: __ __ Inches: __ __

🞏_2_ Don’t know

**17. What was your weight without shoes before you became pregnant?**

🞏_1_ Pounds: __ __ __

🞏_2_ Don’t know

**18. How much did you weigh at age 18?**

🞏_1_ Pounds: __ __ __

🞏_2_ Don’t know

**19. During the past 7 days, on how many days did you walk fast enough to cause your heart rate to increase somewhat for at least 10 minutes at a time?**

🞏_1_ None 🞏_2_ 1-2 🞏_3_ 3-4 🞏_4_ 5-6 🞏_5_ Everyday

🡺 **IF no walking, skip to question 20**

**a. On average, how many minutes did you spend walking each day you walked?**

🞏_1_ 10-19 🞏_2_ 20-29 🞏_3_ 30-59 🞏_4_ 60 or more

**20. During the past 7 days, on how many days did you do other physical activity that required moderate physical effort and caused your heart rate to increase somewhat for at least 10 minutes at a time?**

🞏_1_ None 🞏_2_ 1-2 🞏_3_ 3-4 🞏_4_ 5-6 🞏_5_ Everyday

🡺 **IF no moderate activity, skip to question 21**

**a. On average, how many minutes did you spend doing other physical activity each day you did it?**

🞏_1_ 10-19 🞏_2_ 20-29 🞏_3_ 30-59 🞏_4_ 60 or more

**21. During the past 7 days, on how many days did you do exercise, sports or recreational physical activity for at least 10 minutes at a time that was vigorous enough to work up a sweat or cause your heart rate to increase substantially?**

🞏_1_ None 🞏_2_ 1-2 🞏_3_ 3-4 🞏_4_ 5-6 🞏_5_ Everyday

🡺 **IF no vigorous activity, skip to question 22**

**a. On average, how many minutes did you spend doing vigorous physical activity each day you did it?**

🞏_1_ 10-19 🞏_2_ 20-29 🞏_3_ 30-59 🞏_4_ 60 or more

**22. During the past 7 days, how much time per day, outside of work, did you spend watching TV, videos, or DVDs, using a computer, reading, or driving or riding in a car or other vehicle?**

🞏_1_ Less than 1 hour 🞏_3_ 3 hours up to 5 hours

🞏_2_ 1 hour up to 3 hours 🞏_4_ More than 5 hours

**23. For each of the following foods or beverages, how often do you eat or drink them?** *Using the 12 months before you became pregnant as a guide, please mark how often you usually ate or drank each of these foods.*

| **Food, food group, or beverage** | **I don’t eat or drink**  **this food** | **I eat or drink this food:** | | | | |
| --- | --- | --- | --- | --- | --- | --- |
|  |  | Less than once/ week | 1-2 times/ week | 3-4 times/ week | 5-6 times/ week | Every day |
| Vegetables |  |  |  |  |  |  |
| Fruits |  |  |  |  |  |  |
| Milk (whole, low-fat or skim) |  |  |  |  |  |  |
| Other dairy products (e.g., hard cheese, butter, ice-cream, yogurt, cottage cheese) |  |  |  |  |  |  |
| Whole eggs |  |  |  |  |  |  |
| Margarine (stick-type, not tub) |  |  |  |  |  |  |
| Whole grain foods (e.g., whole grain breads, brown rice) |  |  |  |  |  |  |
| Pasta, rice, noodles |  |  |  |  |  |  |
| Baked products (e.g., donuts, cookies, muffins, crackers, cakes, sweet rolls, pastries) |  |  |  |  |  |  |
| Beans |  |  |  |  |  |  |
| Nuts, seeds, Peanut butter |  |  |  |  |  |  |
| Beef, pork or lamb as main dish |  |  |  |  |  |  |
| Processed meats (sausages, salami, bologna, hot dogs, bacon) |  |  |  |  |  |  |
| Poultry (chicken, turkey, etc.) |  |  |  |  |  |  |
| Fish/seafood |  |  |  |  |  |  |
| Deep fried foods (deep fried chicken, fish or seafood; French fries, onion rings) |  |  |  |  |  |  |
| Caffeinated coffee |  |  |  |  |  |  |
| Black/green tea |  |  |  |  |  |  |
| Soft drinks |  |  |  |  |  |  |

**24. For each of the following vitamins or supplements, how often do you take them?** *Using the 12 months before you became pregnant as a guide, please mark how often you usually took each of these vitamins or supplements.*

| **Type of Vitamin or Supplement** | **I don’t take this vitamin** | **I take this vitamin:** | | | | |
| --- | --- | --- | --- | --- | --- | --- |
|  |  | Less than once/ week | 1-2 times/ week | 3-4 times/ week | 5-6 times/ week | Every day |
| Vitamin C |  |  |  |  |  |  |
| Vitamin D |  |  |  |  |  |  |
| Vitamin E |  |  |  |  |  |  |
| Multivitamins |  |  |  |  |  |  |
| Calcium |  |  |  |  |  |  |
| Iron |  |  |  |  |  |  |
| Selenium |  |  |  |  |  |  |

**25. In the past 2 years, how many of your prescriptions have you filled at a Kaiser Permanente pharmacy?**

🞏_1_ All (100%) 🞏_5_ Very few (1 - 20%)

🞏_2_ Almost all (81 - 99%) 🞏_6_ No prescriptions

🞏_3_ Most (51 - 80%) 🞏_7_ Don’t know

🞏_4_ Some (21- 50%)

**26. Have you ever had a bad reaction (or side effect) to a prescription medication that was serious enough that you had to go to the doctor or hospital for treatment of the reaction?**

🞏_1_ No 🞏_2_ Yes 🞏_3_ Don’t know

🡺What is the name of the medication? _____________

**27. Do you have any allergies?**

*Mark all that apply.*

🞏_1_ Food allergies (e.g., shellfish, nuts)

🞏_2_ Grasses, pollen or dust

🞏_3_ Pets

🞏_4_ Insect stings or bites

🞏_5_ Common medications (e.g., penicillin)

🞏_6_ No known allergies

**28. In the past year, have you had pain that wouldn’t go away (chronic pain)?**

🞏_1_ None of the time 🞏_4_ Most of the time

🞏_2_ A little of the time 🞏_5_ All of the time

🞏_3_ Some of the time

**29. Has a doctor or other health care provider ever told you that you have any of the following medical conditions?** *If yes, please specify the age at which you were first told. Please tell us also if a family member (father, mother, brothers, sisters or children) has or had the condition.*

| **Medical Condition** | **Do you have this condition?** | | **Your age when**  **told** | **Does, or did, a family member have this condition?**  **🞏 If you are adopted, please check this box and skip** | |
| --- | --- | --- | --- | --- | --- |
| Diabetes – Type 1 | 🞏_1_ No | 🞏_2_Yes | **__ __** | 🞏_1_ No | 🞏_2_Yes |
| Diabetes – Type 2 | 🞏_1_ No | 🞏_2_Yes | **__ __** | 🞏_1_ No | 🞏_2_Yes |
| Gestational diabetes  (diagnosed during pregnancy) | 🞏_1_ No | 🞏_2_Yes | **__ __** | 🞏_1_ No | 🞏_2_Yes |
| Angina/Heart attack | 🞏_1_ No | 🞏_2_Yes | **__ __** | 🞏_1_ No | 🞏_2_Yes |
| Congestive heart failure | 🞏_1_ No | 🞏_2_Yes | **__ __** | 🞏_1_ No | 🞏_2_Yes |
| Stroke | 🞏_1_ No | 🞏_2_Yes | **__ __** | 🞏_1_ No | 🞏_2_Yes |
| Multiple Sclerosis | 🞏_1_ No | 🞏_2_Yes | **__ __** | 🞏_1_ No | 🞏_2_Yes |
| Crohn’s disease or ulcerative colitis | 🞏_1_ No | 🞏_2_Yes | **__ __** | 🞏_1_ No | 🞏_2_Yes |
| Systemic Lupus Erythematosus (SLE) | 🞏_1_ No | 🞏_2_Yes | **__ __** | 🞏_1_ No | 🞏_2_Yes |
| Depression | 🞏_1_ No | 🞏_2_Yes | **__ __** | 🞏_1_ No | 🞏_2_Yes |
| Bipolar disorder | 🞏_1_ No | 🞏_2_Yes | **__ __** | 🞏_1_ No | 🞏_2_Yes |
| Panic disorder | 🞏_1_ No | 🞏_2_Yes | **__ __** | 🞏_1_ No | 🞏_2_Yes |
| Asthma | 🞏_1_ No | 🞏_2_Yes | **__ __** | 🞏_1_ No | 🞏_2_Yes |
| Cancer  If yes, what kind(s)?  ___________________  ___________________ | 🞏_1_ No | 🞏_2_Yes | **__ __** | 🞏_1_ No | 🞏_2_Yes |
| COPD or Emphysema | 🞏_1_ No | 🞏_2_Yes | **__ __** | 🞏_1_ No | 🞏_2_Yes |
| ALS or Lou Gehrig’s disease | 🞏_1_ No | 🞏_2_Yes | **__ __** | 🞏_1_ No | 🞏_2_Yes |
| Parkinson’s disease | 🞏_1_ No | 🞏_2_Yes | **__ __** | 🞏_1_ No | 🞏_2_Yes |
| Dementia/ Alzheimer’s disease | 🞏_1_ No | 🞏_2_Yes | **__ __** | 🞏_1_ No | 🞏_2_Yes |
| Dystonia, torticollis, blepharospasm | 🞏_1_ No | 🞏_2_Yes | **__ __** | 🞏_1_ No | 🞏_2_Yes |
| Schizophrenia or other psychosis | 🞏_1_ No | 🞏_2_Yes | **__ __** | 🞏_1_ No | 🞏_2_Yes |
| HIV/AIDS | 🞏_1_ No | 🞏_2_Yes | **__ __** | 🞏_1_ No | 🞏_2_Yes |
| Chronic hepatitis | 🞏_1_ No | 🞏_2_Yes | **__ __** | 🞏_1_ No | 🞏_2_Yes |
| Polycystic ovary syndrome (PCOS) | 🞏_1_ No | 🞏_2_Yes | **__ __** | 🞏_1_ No | 🞏_2_Yes |
| Hypertension/high blood pressure | 🞏_1_ No | 🞏_2_Yes | **__ __** | 🞏_1_ No | 🞏_2_Yes |
| Anorexia nervosa or Bulimia | 🞏_1_ No | 🞏_2_Yes | **__ __** | 🞏_1_ No | 🞏_2_Yes |
| Restless leg syndrome | 🞏_1_ No | 🞏_2_Yes | **__ __** | 🞏_1_ No | 🞏_2_Yes |
| Rheumatoid arthritis | 🞏_1_ No | 🞏_2_Yes | **__ __** | 🞏_1_ No | 🞏_2_Yes |
| Other arthritis | 🞏_1_ No | 🞏_2_Yes | **__ __** | 🞏_1_ No | 🞏_2_Yes |
| Osteoporosis | 🞏_1_ No | 🞏_2_Yes | **__ __** | 🞏_1_ No | 🞏_2_Yes |
| Heartburn or acid regurgitation | 🞏_1_ No | 🞏_2_Yes | **__ __** | 🞏_1_ No | 🞏_2_Yes |

**30. On average, how many days a week did you have a drink containing alcohol before you became pregnant?**

🞏_1_ No days 🞏_5_ 4 days

🞏_2_ 1 day 🞏_6_ 5 days

🞏_3_ 2 days 🞏_7_ 6 days

🞏_4_ 3 days 🞏_8_ Everyday

**31. In the year before you became pregnant, on a typical day that you drank, how many drinks containing alcohol did you have?**

🞏_1_ didn’t drink 🞏_2_ 1 🞏_3_ 2 🞏_4_ 3 🞏_5_ 4 🞏_6_ 5 🞏_7_ 6 🞏_8_ 7 🞏_9_ 8 or more

**32. How many times in the year before you became pregnant have you had four or more drinks containing alcohol in a day*?*** *(One standard drink is 12 ounces of beer, 5 ounces of wine, or a one-ounce shot of liquor.)*

🞏_1_ More than twice monthly (25 or more times)

🞏_2_ Once or twice monthly (12-24 times)

🞏_3_ Less than monthly (1-11 times)

🞏_4_ I never drink 4 or more drinks in a day

**33. Have you ever smoked one or more *cigarettes* per day for six months or longer?**

🞏_1_ No 🞏_2_ Yes

**If No, please skip to question 34**

🡺 **Do you currently smoke or have you stopped smoking?**

🞏_1_ Current smoker

🞏_2_ Former smoker

**🡺 For about how many years have you smoked (or did you smoke) cigarettes altogether? (Do not count periods where you were not smoking or had quit.)**

🞏_1_ 1-5 years 🞏_3_ 11-16 years

🞏_2_ 6-10 years 🞏_4_ 16 or more years

**🡺 On average, how many packs of cigarettes do you (or did you) smoke per day?**

🞏_1_ less than ½ pack

🞏_2_ ½ - 1 pack

🞏_3_ 1- 1 ½ packs

🞏_4_ more than 1½ packs

**34. At what age did you have your first menstrual period?**

🞏_1_ Less than 10 years

🞏_2_ 10-11 years 🞏_5_ 16 years or older

🞏_3_ 12-13 years 🞏_6_ Don’t know

🞏_4_ 14-15 years 🞏_7_ Never had a menstrual period

**35. In the year before you became pregnant, what best describes your menstrual cycle?**

🞏_1_ I had regular periods

🞏_2_ I had irregular periods

🞏_3_ I had periods because I took hormones

🞏_4_ I didn’t have a period

**🡺 Why?**

🞏_1_ Recent pregnancy/breastfeeding

🞏_2_ Medical treatment

🞏_3_ Don’t know

**36. Including your current pregnancy, how many times have you been pregnant? Please include miscarriages, stillbirths, tubal or ectopic pregnancies, abortions and livebirths.**

🞏_1_ One 🞏_2_ Two 🞏_3_ Three 🞏_4_ Four or more

**a. How many children have you given birth to?**

🞏_1_ None 🞏_2_ One 🞏_3_ Two 🞏_4_ Three 🞏_5_ Four or more

**IF you marked None please skip to question 37**

**b. How old were you when you gave birth for the first time?**

🞏_1_ Less than 16 years 🞏_5_ 30-34 years

🞏_2_ 16-19 years 🞏_6_ 35-39 years

🞏_3_ 20-24 years 🞏_7_ 40 years or older

🞏_4_ 25-29 years 🞏_8_ Don’t know

**37. Please tell us who completed this survey.**

🞏_1_ The person to whom the survey packet was addressed

🞏_2_ Person to whom the survey packet was addressed, with help from someone else

🞏_3_ Spouse/partner of the person to whom the packet was addressed

🞏_4_ Other (specify):__________________

**Today’s Date 🞎🞎/ 🞎🞎 /🞎🞎🞎🞎**

**MO / DAY / YEAR**

*Thanks for taking the time to complete this survey*
